# Kidney-targeted drug delivery: from physiological mechanisms to precision therapeutics

**DOI:** 10.3389/fbioe.2026.1763546

**Published:** 2026-04-02

**Authors:** Zhanyi Tan, Yuan Hu, Yajun Wu, Yan Hu

**Affiliations:** 1 Department of Pediatric Urinary Disease Centre Nursing, West China Second University Hospital, Sichuan University/West China School of Nursing, Sichuan University, Chengdu, Sichuan, China; 2 Key Laboratory of Birth Defects and Related Diseases of Women and Children (Sichuan University), Ministry of Education, Chengdu, Sichuan, China; 3 Department of Pediatric Cardiology Nursing, West China Second University Hospital, Sichuan University/West China School of Nursing, Sichuan University, Chengdu, Sichuan, China; 4 West China Hospital, Sichuan University/West China School of Nursing, Sichuan University, Chengdu, Sichuan, China

**Keywords:** acute kidney injury, chronic kidney disease, glomerular filtration barrier, kidney-targeted drug delivery, nanoparticles, prodrugs, Renal tubules

## Abstract

Chronic kidney disease (CKD) and acute kidney injury (AKI) remain critical global health challenges, yet effective pharmacotherapies are severely limited by poor renal bioavailability and off-target systemic toxicity. Overcoming these obstacles requires a deep integration of renal physiopathology with advanced drug delivery engineering. This review provides a comprehensive analysis of the mechanisms governing kidney-targeted drug delivery. We first dissect the unique physiological barriers that dictate renal drug disposition, including the size- and charge-selective glomerular filtration barrier (“the sieve”) and the high-capacity reabsorption machinery of the proximal tubule (e.g., megalin-mediated endocytosis). Subsequently, we elucidate the pathological alterations in the “fibrotic niche”, highlighting emerging therapeutic targets such as the upregulated CD44 receptor and specific integrins on myofibroblasts. Based on this understanding, we systematically categorize current delivery strategies into two paradigms: (1) Passive Targeting, which exploits physicochemical properties (e.g., 75–100 nm size range for mesangial sequestration); and (2) Active Targeting, which utilizes ligand-receptor precision to direct carriers to the tubular epithelium (via megalin or transporters) or the fibrotic microenvironment (via hyaluronic acid or RGD [Arg-Gly-Asp]). Finally, we discuss the challenges of clinical translation, including interspecies differences and long-term nanotoxicology, and outline future directions in bio-inspired vectors and stimuli-responsive logic-gated systems. Ultimately, the seamless integration of these modular delivery platforms with patient-specific molecular signatures heralds a new era of precision nephrology, moving beyond systemic management toward site-specific interventions that may fundamentally reverse the progression of renal failure.

## 1 Introduction

The kidney serves as the physiological cornerstone of internal homeostasis, filtering approximately 180 L of plasma filtrate daily to eliminate metabolic waste while reclaiming 99% of water and essential solutes (Koeppen BM, 2012; Felmlee et al., 2013). This organ’s function is sustained by a complex vascular and tubular architecture, making it indispensable for regulating blood pressure, electrolyte balance, and erythropoiesis (Romagnani et al., 2025). However, this high metabolic activity and vascular exposure render the kidney exceptionally vulnerable to insults. The global burden of kidney diseases—ranging from reversible acute kidney injury (AKI) to progressive chronic kidney disease (CKD)—is escalating rapidly, with CKD alone affecting over 8% of the global population and significantly increasing cardiovascular mortality (Kellum et al., 2021; Levey and James, 2017; Chen et al., 2019). Regardless of the initial aetiology, persistent renal injury invariably triggers a cascade of inflammation, capillary rarefaction, and extracellular matrix deposition, ultimately leading to renal fibrosis and end-stage renal disease (Li et al., 2022; Venkatachalam et al., 2015).

Despite the urgent clinical need, effective pharmacological intervention is severely hampered by the kidney’s intrinsic anatomical barriers. The renal parenchyma is protected by the glomerular filtration barrier (GFB), a highly selective sieve composed of fenestrated endothelium, the glomerular basement membrane (GBM), and epithelial podocytes (Miner, 2012). This barrier strictly regulates the passage of molecules based on size and charge, effectively preventing most conventional macromolecular therapeutics from reaching glomerular targets such as the mesangium or podocytes (Huang et al., 2021). Furthermore, while the renal proximal tubules possess robust transport machinery (e.g., megalin and cubilin receptors) for reabsorbing nutrients, they differentiate poorly between therapeutic agents and toxic xenobiotics, often limiting drug retention time or leading to unintended tubular toxicity (De et al., 2014; Williams et al., 2018).

Current standard-of-care treatments, including corticosteroids, immunosuppressants, and renin-angiotensin-aldosterone system inhibitors, rely predominantly on systemic administration (Natale et al., 2022). This “untargeted” approach presents a fundamental therapeutic dilemma: achieving effective drug concentrations within fibrotic lesions or inflamed glomeruli often requires high systemic doses, which inevitably precipitate severe off-target adverse effects, such as hepatotoxicity, gastrointestinal ulceration, and bone marrow suppression (Shang et al., 2024; Zhou et al., 2014). Moreover, as renal pathology progresses, maladaptive repair mechanisms and glomerulosclerosis further impede the diffusive transport of drugs into the injured tissue, rendering systemic therapy increasingly inefficient (Perico et al., 2008; Li et al., 2023).

Consequently, the development of kidney-targeted drug delivery systems (KDDS) represents a paradigm shift in nephrology (Kamaly et al., 2016). This ongoing transformation, as highlighted in recent 2025–2026 perspectives, moves beyond conventional systemic management toward site-specific precision interventions that integrate advanced engineering principles with emerging renoprotective strategies (Wang et al., 2026; Bazargani, 2025). By engineering carriers that specifically exploit renal physiological features—such as size-dependent entrapment in the mesangium or ligand-receptor mediated endocytosis in tubular cells—therapeutics can be delivered with high precision (Liu et al., 2019; Dolman et al., 2010). This review critically examines the physiological and pathological considerations for carrier design. It categorises emerging targeting strategies, including low-molecular-weight protein conjugates, prodrugs, and nanomedicines, discussing their potential to overcome biological barriers and arrest the progression of renal disease (Yu et al., 2021).

## 2 Physiopathology of the kidney

**FIGURE 1:**
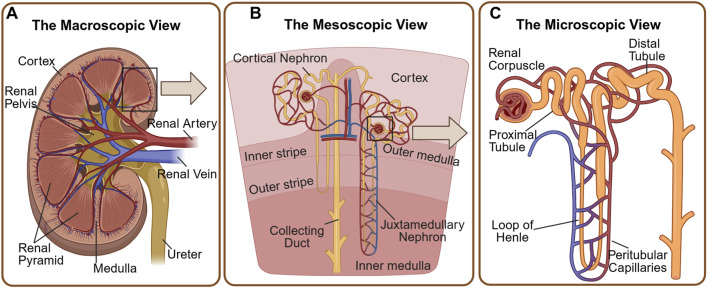
Anatomical organisation of the kidney: from organ to nephron. **(A)** Macroscopic architecture: A longitudinal section of the kidney illustrates the distinct physiological zones: the outer cortex, which houses the glomeruli and proximal tubules, and the inner medulla (renal pyramids), responsible for the osmotic gradient. The renal pelvis and major vasculature (renal artery and vein) are also shown. **(B)** Mesoscopic arrangement: This view highlights the spatial organisation of the nephrons within the parenchyma. Cortical nephrons are situated superficially with short loops of Henle. In contrast, juxtamedullary nephrons lie near the corticomedullary junction with long loops penetrating deep into the medulla, a feature critical for urine concentration. The collecting ducts traverse these zones to drain urine into the pelvis. **(C)** The microscopic functional unit: a schematic of a single nephron details its segmental compartmentalisation. The renal corpuscle initiates blood filtration. The renal tubule is divided into the proximal tubule (the primary hub for reabsorption), the loop of Henle, and the distal tubule. The peritubular capillaries maintain a close spatial relationship with the tubules to facilitate the exchange of reabsorbed nutrients and secreted xenobiotics.

The kidney is a vital organ in the human urinary system, primarily responsible for maintaining physiological stability through blood filtration, homeostatic regulation, and the excretion of metabolic waste products (Koeppen BM, 2012). This functional complexity is supported by millions of intricate units, known as nephrons, which serve as the core structural components that sustain these critical processes (Figure 1). Anatomically, each nephron is composed of a renal corpuscle--encompassing Bowman’s capsule and the glomerular network of blood vessels--and an associated system of renal tubules, which together orchestrate the filtration, reabsorption, and secretion of fluids and blood constituents (Nørregaard et al., 2023; Sethi and Fervenza, 2025).

However, under pathological conditions, disregulation of these renal functions can precipitate a spectrum of severe disorders, ranging from AKI, characterised by a sudden drop in glomerular filtration rate, to CKD, which involves irreversible nephron loss (Ostermann et al., 2025; Kovesdy, 2025). Therefore, comprehending these intricate physiological and pathological traits is an imperative prerequisite for developing kidney-targeted drug delivery systems. Such systems are essential for enhancing drug concentrations in specific renal cells, thereby optimising therapeutic outcomes while mitigating the severe systemic adverse reactions often associated with high-dose, non-targeted administration (Huang et al., 2021; Shang et al., 2024).

### 2.1 The glomerulus

The glomerulus constitutes the kidney’s primary filtration unit, receiving approximately 20% of the cardiac output. Its physiological function is maintained by the GFB, a highly sophisticated biological sieve that strictly regulates the passage of plasma components based on size and charge. Recent ultrastructural analyses have refined our understanding of the GFB, proposing a “five-layer” model that includes the endothelial surface layer and the subpodocyte space, in addition to the classic three layers (Altintas et al., 2025).

#### 2.1.1 The multilayered filtration assembly

**FIGURE 2:**
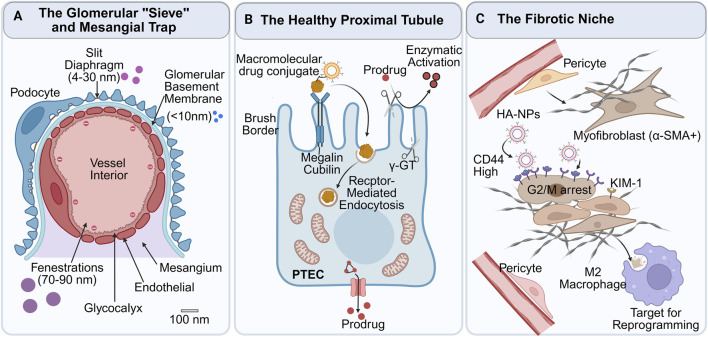
Schematic illustration of physiological barriers and pathological targets in the kidney. **(A)** The GFB functions as a size- and charge-selective sieve. Nanoparticles sized 75–100 nm are passively sequestered in the mesangial niche, while smaller molecules are filtered. **(B)** The healthy proximal tubule utilises physiological reabsorption machinery for active targeting. Macromolecular carriers “hitchhike” on the megalin/cubilin receptor-mediated endocytic pathway, while prodrugs are activated by brush-border enzymes (e.g., γ-GT) or transported via OAT/OCT channels. **(C)** The fibrotic niche: during maladaptive repair, tubular cells undergo pEMT, characterised by the loss of brush border and the upregulation of disease-specific receptors (CD44, KIM-1). Targeted carriers (e.g., HA-conjugates) exploit these markers, while stromal targets include pericyte-to-myofibroblast transition and macrophage polarisation.

The GFB constitutes a highly sophisticated biological sieve that strictly regulates the passage of plasma components based on size and charge (Figure 2A). While traditionally described as a three-layered structure, recent ultrastructural analyses have refined this model to emphasise the functional dominance of the endothelial glycocalyx and the podocyte slit diaphragm in drug delivery selectivity (Altintas et al., 2025; Balbotkina and Kutina, 2023).

The innermost barrier, the endothelial surface layer, comprises fenestrated glomerular endothelial cells with pores measuring 70–90 nm in diameter. However, recent physiological data indicate that these fenestrations are filled with a thick (200–400 nm), negatively charged glycocalyx. This gel-like layer serves as the primary determinant of charge selectivity, effectively repelling anionic proteins and negatively charged drug carriers. Consequently, novel nanomedicines designed with neutral or slightly positive surface charges demonstrate optimised penetration by avoiding the immediate entrapment observed with highly cationic carriers, which are often repelled before reaching the basement membrane (Li et al., 2023).

Beneath the endothelium lies the GBM, a dense, acellular meshwork of extracellular matrix proteins—primarily laminin-521 and type IV collagen—typically measuring 9–10 nm in mesh width. The GBM functions as a robust size-selective sieve and provides mechanical resistance against filtration pressure. While it contains anionic proteoglycans like heparan sulfate, emerging evidence suggests its role is more structural, restricting the passage of macromolecules larger than 10 nm regardless of charge (Li et al., 2023).

The final and most critical barrier consists of podocytes, specialised postmitotic epithelial cells that wrap around capillaries. Their interdigitating foot processes form filtration slits ranging from 4 to 30 nm, bridged by the slit diaphragm protein complex, which contains nephrin and podocin. Podocyte injury, characterised by foot process effacement and cytoskeleton rearrangement, is the central pathogenic mechanism in focal segmental glomerulosclerosis. Since podocytes possess limited regenerative capacity, they represent a critical yet vulnerable target. Recent reviews highlight that targeting podocyte-specific receptors, such as the neonatal Fc receptor (FcRn) or integrins, is essential for next-generation nanotherapies to treat proteinuric kidney diseases (Meliambro et al., 2024).

#### 2.1.2 The mesangial niche

The glomerular mesangium, located between the capillary loops, serves as the central structural support for the glomerulus and plays a pivotal role in regulating glomerular hemodynamics. Unlike the peripheral capillary wall, the mesangium lacks a complete basement membrane, creating a unique “window” for particle entry. This anatomical feature allows for a highly efficient passive targeting strategy known as “size-dependent mesangial entrapment”.

Recent quantitative analyses confirm that this entrapment is strictly governed by particle diameter. Nanoparticles (NPs) within the specific size range of 75–100 nm are small enough to pass through the endothelial fenestrations (70–90 nm) but too large to cross the mesangial matrix or enter the tubular lumen (Choi et al., 2011). Consequently, these particles become sequestered in the mesangial space for extended periods. This phenomenon is particularly advantageous for treating mesangial proliferative diseases, such as immunoglobulin A (IgA) nephropathy and diabetic nephropathy. In these conditions, activated mesangial cells proliferate rapidly and secrete proinflammatory cytokines and excess extracellular matrix, driving glomerulosclerosis (Xiong et al., 2025).

Targeting this niche has shown remarkable therapeutic potential. A 2025 study highlighted that IgA autoantibodies naturally target mesangial antigens, specifically beta-II spectrin (βII-spectrin)—a critical cytoskeletal protein involved in maintaining cell membrane integrity—thereby providing a biological blueprint for designing targeted carriers (Nihei et al., 2023). Building on this, researchers have engineered celastrol-loaded albumin nanoparticles (∼95 nm) designed to leverage the optimal physiological window for mesangial sequestration. These nanoparticles effectively localised in mesangial cells, suppressing proliferation and matrix deposition in rat models of glomerulonephritis while significantly reducing the systemic toxicity associated with free celastrol (Guo et al., 2017). Furthermore, surface charge plays a critical modulatory role: anionic nanoparticles are preferentially retained in the mesangium due to interactions with scavenger receptors, whereas cationic particles are cleared more rapidly (Shang et al., 2024). However, while these rodent models provide essential proof-of-concept data, clinical translation must account for potential interspecies divergence in mesangial matrix density and glomerular hemodynamics, which may significantly alter the long-term sequestration of 95-nm mesoscale vehicles in human patients compared to observed murine kinetics.

#### 2.1.3 Glomerular molecular targets in inflammation

While the filtration barrier and mesangium represent physical obstacles, the glomerular endothelium and podocytes express specific molecular targets during pathological states, offering opportunities for active targeting. Unlike the quiescent endothelium in healthy kidneys, glomerular endothelial cells in diseases such as lupus nephritis and vasculitis undergo “activation.”

A hallmark of this activation is the dramatic upregulation of cell adhesion molecules (CAMs). Recent transcriptional profiling in 2024 has confirmed that E-selectin and vascular cell adhesion molecule-1 (VCAM-1) are significantly overexpressed on the surface of inflamed glomerular endothelial cells (Cavanaugh and Okusa, 2021). These molecules, which normally recruit leukocytes, can serve as “docking stations” for ligand-modified nanocarriers. For instance, drug carriers conjugated with anti-E-selectin antibodies can selectively bind to the inflamed glomerular endothelium, resisting the shear stress of blood flow (Shang et al., 2024; Hu et al., 2025).

Furthermore, podocytes present unique receptor targets hidden behind the GBM. The neonatal FcRn, which is highly expressed in human podocytes, has emerged as a critical portal. FcRn functions to recycle albumin and immunoglobulin G (IgG), preventing their lysosomal degradation. Harnessing this pathway with albumin-based or Fc-fused therapeutics enables active transport of drugs into podocytes, bypassing the size restrictions of the slit diaphragm (Meliambro et al., 2024). Understanding these inducible surface markers is a prerequisite for designing the “next-generation” of glomerular-targeted therapies that go beyond simple size-dependent entrapment.

### 2.2 The renal tubules

The renal tubular system, a highly segmented continuum extending from Bowman’s capsule to the collecting duct, acts as the kidney’s metabolic engine. While often simplified as a single unit, the tubules exhibit distinct segmental functions—filtration and reabsorption in the proximal tubule, concentration in the loop of Henle, and fine-tuning in the distal segments—each with unique molecular targets for drug delivery (Figure 1C).

#### 2.2.1 The proximal tubule

The proximal tubule, extending directly from the urinary pole of the glomerulus, represents the most metabolically active and structurally complex segment of the nephron, functionally tasked with the “bulk reclamation” of the glomerular filtrate. Physiologically, this segment is responsible for reabsorbing approximately 65% of filtered water and electrolytes, along with virtually all filtered glucose and amino acids, via a combination of active and passive transport mechanisms (Felmlee et al., 2013; Romagnani et al., 2025). To support this immense transport workload, proximal tubular epithelial cells (PTECs) exhibit a highly specialised ultrastructure, characterised by a dense mitochondrial population and a prominent apical brush border. This brush border is composed of extensive microvilli that drastically amplify the absorptive surface area, thereby maximising the interface for solute-carrier interactions (Romagnani et al., 2025). Maintaining this intricate architecture requires a colossal amount of adenosine triphosphate (ATP), derived almost exclusively from aerobic oxidative phosphorylation. Consequently, PTECs are obligate aerobes with limited glycolytic capacity, making them exquisitely sensitive to hypoxic insults and mitochondrial dysfunction (Venkatachalam et al., 2015). This metabolic vulnerability underpins the pathogenesis of AKI, where ischemic insults or nephrotoxins (such as cisplatin or aminoglycosides) lead to rapid ATP depletion, cytoskeletal breakdown, and necrosis of the tubular epithelium (Chatterjee and Zarjou, 2025; Wang Y. et al., 2024). Understanding this “metabolic engine” is crucial for drug delivery, as the high density of mitochondria provides a specific subcellular target for emerging antioxidant therapies aimed at mitigating acute renal injury (Chatterjee and Zarjou, 2025).

While the glomerular barrier restricts the passage of large macromolecules, the proximal tubule possesses a sophisticated array of transporters and metabolic enzymes that govern the disposition of small molecules and xenobiotics. The basolateral and apical membranes of PTECs are studded with polyspecific transporters, including those for organic anions and cations, which facilitate the secretion of waste products and the reabsorption of nutrients (Wagner, 2025). This transport machinery serves as a double-edged sword: while it is essential for homeostasis, it also functions as an “entry gate” for nephrotoxic accumulation. For instance, the specific accumulation of cisplatin in PTECs is driven by active transport, leading to cellular toxicity (Wagner, 2025). However, this same machinery offers a pivotal opportunity for “active targeting” via prodrug strategies. The proximal tubule is uniquely rich in membrane-bound metabolic enzymes, such as γ-glutamyl transpeptidase (γ-GT) and aromatic L-amino acid decarboxylase, which are predominantly localised at the brush border (Wilk et al., 1978). By chemically modifying parent drugs with specific amino acids (e.g., glutamate), researchers have developed prodrugs like gamma-glutamyl-dopa (Dolman et al., 2010). These prodrugs remain inert in systemic circulation but are specifically hydrolysed by renal brush-border enzymes, releasing the active dopamine payload exclusively within the kidney to act as a renal vasodilator (Pestana and Soares-da-Silva, 1994). This “metabolic targeting” strategy effectively leverages the unique enzymatic profile of the PT to achieve high local drug concentrations while minimising systemic adverse effects such as hypotension or hyperglycemia (Zhou et al., 2014; Su et al., 2003).

For macromolecules, polypeptides, and nanoparticles that successfully pass through the glomerular filtration barrier, the proximal tubule employs a high-capacity, receptor-mediated endocytic system acting as a “scavenger pathway.” This process is primarily orchestrated by the giant multiligand receptors megalin and cubilin, which are abundantly expressed at the apical clathrin-coated pits of PTECs (De et al., 2014; Nielsen et al., 2016). These receptors bind to a vast array of ligands—including albumin, low-molecular-weight proteins, and polybasic drugs—triggering rapid invagination and endosomal delivery. Recent studies confirm that megalin undergoes rapid recycling, creating a continuous “conveyor belt” for internalisation (Kulkarni and Hussain, 2025). This pathway serves as the foundation for macromolecular kidney targeting. Endogenous low-molecular-weight proteins (LMWPs) such as lysozyme (<30 kDa) are natural substrates for megalin and are extensively reabsorbed by proximal tubular cells (Haas et al., 1997; Jaspers et al., 2025). Consequently, conjugating drugs to lysozyme or using polymers that mimic ligand characteristics, such as Low Molecular Weight Chitosan (LMWC), allows therapeutics to “hitchhike” on this recycling route (Li et al., 2023; Yuan et al., 2009). Studies have confirmed that LMWC uptake is closely linked to megalin-mediated endocytosis, enabling the specific internalisation of siRNA or anti-fibrotic agents into PTECs (Liu et al., 2019; Gao et al., 2014). However, the design of such nanocarriers requires careful optimisation; excessive delivery can saturate the megalin pathway or overwhelm lysosomal degradation capacity, potentially inducing tubular toxicity (Kulkarni and Hussain, 2025). Thus, exploiting this endocytic gateway represents a promising yet delicate balance for treating tubular atrophy and renal fibrosis (Dolman et al., 2010; Williams et al., 2015).

#### 2.2.2 The fibrotic niche: maladaptive repair and tubular phenotypic switching

While the proximal tubule possesses a remarkable intrinsic capacity for regeneration following mild ischemic or toxic insults, severe, recurrent, or sustained injury often exceeds this reparative threshold (Venkatachalam et al., 2015). This failure of restitution triggers a pathological cascade known as maladaptive repair, which is now recognised as the central biological driver of the transition from AKI to CKD (He et al., 2017). Instead of restoring a healthy, polarised epithelium, a subpopulation of injured PTECs undergoes cell cycle arrest, specifically stalling in the G2/M phase (Tang et al., 2020). These arrested cells become metabolically active but non-proliferative, adopting a Senescence-Associated Secretory Phenotype (SASP)2. Functioning as proinflammatory “factories,” they release a profusion of profibrotic cytokines--including Transforming Growth Factor-β1 (TGF-β1), Connective Tissue Growth Factor (CTGF), and Interleukin-6 (IL-6)-into the surrounding interstitium (Romagnani et al., 2025). This “cytokine storm” activates resident pericytes and fibroblasts, stimulating their transdifferentiation into myofibroblasts and accelerating the deposition of extracellular matrix (ECM) components, such as collagen I/III and fibronectin (Li et al., 2022; Tang et al., 2020).

Recent breakthroughs in spatial transcriptomics (2024) have redefined our understanding of this process. A landmark study by Abedini et al. (published in Nature Genetics, 2024) utilised single-cell multi-omic profiling to map the human kidney, identifying a distinct “Fibrotic Microenvironment” (Figure 2C). This niche is not merely a collection of scar tissue but a highly organised “neighbourhood” where maladaptive tubules, VCAM1+ endothelial cells, and myofibroblasts physically co-localise. Within this niche, injured tubules lose their physiological identity and act as signalling hubs that perpetuate inflammation (Abedini et al., 2024).

A defining feature of this fibrotic transformation is a process termed partial Epithelial-Mesenchymal Transition (pEMT) (Di et al., 2025). Unlike fully EMTed cells observed in embryogenesis or cancer metastasis, tubular cells in pEMT do not migrate away to become fibroblasts; instead, they remain integrated within the tubular basement membrane and undergo a dramatic phenotypic switch (Romagnani et al., 2025). During pEMT, injured PTECs lose their apical-basal polarity and downregulate key epithelial adhesion molecules such as E-cadherin, while simultaneously acquiring mesenchymal markers such as Vimentin and α-Smooth Muscle Actin (α-SMA) (Li et al., 2022; Poulsom, 2007). Crucially for drug delivery design, this phenotypic switch is accompanied by a radical alteration in the cell’s transport machinery. The expression of physiological solute carriers—such as Organic Anion Transporters (OATs) and Organic Cation Transporters (OCTs)—is markedly reduced or silenced in fibrotic tubules (Shang et al., 2024). This metabolic downregulation explains why conventional systemic drugs or prodrugs targeting healthy transport pathways often fail to reach therapeutic concentrations in diseased kidneys. Conversely, molecules associated with cell adhesion are upregulated, offering “disease-specific” molecular addresses (Liu et al., 2019).

The most prominent target emerging from this surface remodelling is the CD44 receptor. In the healthy kidney, CD44 expression is minimal or undetectable (Kocak et al., 2009). However, following ischemic or toxic injury, its expression increases nearly 10-fold on the basolateral surface of injured tubular cells and activated macrophages. As the primary receptor for hyaluronan and osteopontin, CD44 mediates the migration of tubular cells on the provisional fibrin matrix during repair. This pathological overexpression provides the physiological rationale for using Hyaluronic Acid (HA)-based prodrugs or nanoparticles. Research has demonstrated that HA-conjugated therapeutics can actively bind to CD44 on injured cells via ligand-receptor interactions. For instance, HA-curcumin conjugates exhibited significantly enhanced internalisation in injured PTECs compared to free curcumin, leveraging the CD44 pathway to deliver anti-inflammatory payloads precisely to the “inflamed” cells while sparing healthy tissue (Hu et al., 2018).

In parallel to CD44, Kidney Injury Molecule-1 (KIM-1) is another high-fidelity target. KIM-1 is virtually absent in normal tubules but becomes the most highly upregulated protein in the injured kidney, transforming the apical membrane into a phagocytic surface. This transformation enables surviving epithelial cells to engulf apoptotic debris. Nanocarriers designed to mimic apoptotic bodies (e.g., via phosphatidylserine exposure) or modified with KIM-1-binding ligands can exploit this phagocytic mechanism to deliver anti-fibrotic payloads directly into the intracellular compartment of the maladaptive cells (Shang et al., 2024).

Finally, the metabolic state of the fibrotic niche offers a novel targeting dimension. Injured tubules undergo a metabolic switch from fatty acid oxidation (FAO) to glycolysis, a phenomenon termed the “Warburg effect”. This shift leads to lipid accumulation and lipotoxicity, further driving fibrosis (Mukhi et al., 2023). New therapeutic strategies in 2025 are focusing on delivering metabolic modulators (such as PPARα agonists) specifically to these lipid-overloaded cells to restore FAO and arrest fibrogenesis.

#### 2.2.3 Transporter

While receptor-mediated endocytosis offers a high-capacity pathway for macromolecules, small-molecule therapeutics can be engineered to “hijack” the kidney’s specific nutrient transport machinery. The renal proximal tubule is equipped with an extensive array of transporters for sugars and vitamins, which are essential for metabolic homeostasis but can also serve as “molecular gates” for targeted drug delivery. The reabsorption of glucose, a critical energy substrate for the kidney, is strictly mediated by sodium-glucose cotransporters (SGLT1 and SGLT2) on the apical membrane and glucose transporters (GLUT1 and GLUT2) on the basolateral membrane (Felmlee et al., 2013; Taub et al., 2010). In pathological states such as diabetic kidney disease and fibrosis, the expression and activity of these transporters are often altered, creating a specific “metabolic vulnerability” that can be exploited for drug entry (Liu et al., 2019; Wagner, 2025). Mechanistically, conjugating drugs to glucose or glucose analogues (glycosylation) allows them to be recognised by SGLTs or GLUTs as pseudo-substrates, thereby facilitating their active transport into the cytoplasm (Suzuki et al., 1997). A classic implementation of this strategy involves an alkylglucoside vector. Pioneering studies by Suzuki et al. demonstrated that glucosylated peptide derivatives, such as Arg8-vasopressin, exhibit specific accumulation in kidney membranes due to high-affinity interactions with glucose transporters (Suzuki et al., 1999a; Wei et al., 2025). The chemical nature of the linkage is crucial; the alkylglucoside structure (e.g., Glc-S-C8-) was identified as a key determinant for renal specificity, with the length of the alkyl chain and the site of conjugation significantly influencing clearance rates (Su et al., 2003; Shirota et al., 2001).

Building on this glucose-targeting platform, recent advancements have focused on enhancing the intracellular retention of anti-inflammatory steroids. Free prednisolone typically diffuses non-specifically, leading to systemic toxicity. To address this, Lin et al. synthesised a prednisolone succinate-glucosamine conjugate that utilises the glucose transport machinery to enter PTECs (Lin et al., 2012a). *In vitro* studies confirmed a 2.2-fold enhancement in drug uptake by kidney cell lines (HK-2) compared to the unmodified drug, without increasing cytotoxicity (Lin et al., 2012a). Further *in vivo* investigations using a 2-deoxy-2-aminodiglucose-prednisolone conjugate (DPC) revealed a 4.9-fold increase in renal drug concentration, validating the potential of aminosugar vectors for targeted delivery (Lin et al., 2012b). More recently, the focus has shifted to the SGLT2 protein itself, not just as a transporter but as a therapeutic docking site. With SGLT2 inhibitors (gliflozins) becoming standard care for CKD, 2025 reviews highlight that SGLT2 modulation also suppresses the Nucleotide-binding oligomerisation domain, Leucine-rich Repeat and Pyrin domain containing 3 (NLRP3) inflammasome in PTECs (Rabbani et al., 2025; Yao et al., 2025). The recognition of SGLT2’s dual role in both metabolic transport and inflammatory regulation has spurred the design of next-generation “sugar-drug conjugates” that aim to bind SGLT2 synergistically, using the transporter for entry while simultaneously blocking its proinflammatory signalling (Yu et al., 2021; Shang et al., 2024).

In parallel with sugar transporters, the vitamin transport system provides another high-fidelity route, particularly via the folate receptor (FR). Folate (Vitamin B9) is essential for DNA synthesis and repair, and its uptake is mediated by the high-affinity FR, a glycosylphosphatidylinositol (GPI)-anchored protein (Lin et al., 2024; Low et al., 2008). In healthy tissues, FR-α expression is primarily restricted to the renal proximal tubules, where it recovers filtered folate. However, in the context of renal fibrosis and polycystic kidney disease (PKD), FR expression becomes pathologically upregulated on the apical surface of proliferating or cystic tubular cells (Perico et al., 2008; Shi et al., 2019). This differential expression makes folate an ideal ligand for targeting the “activated” tubular epithelium. Mathias et al. first demonstrated this potential by showing that Indium-111-Labelled DTPA-folate could effectively target proximal tubules for imaging (Chan et al., 2023). This concept was further advanced by Shi et al., who designed folate-dactolisib conjugates to specifically target the mTOR pathway in cystic renal epithelial cells, effectively slowing cyst growth in PKD models (Shi et al., 2019).

The utility of folate targeting has recently been expanded to nanomedicine, addressing the dual nature of renal inflammation. Beyond tubular cells, the FR-β isoform is significantly overexpressed on activated, profibrotic macrophages infiltrating the kidney (Chatterjee and Zarjou, 2025; Lin et al., 2024). This dual upregulation allows folate-modified carriers to target the “fibro-inflammatory unit” as a whole. A landmark 2024 study reported the development of sub-10-nm folic acid-conjugated gold nanoparticles (FA-AuNPs) (Chan et al., 2023). These ultra-small particles were engineered to pass the glomerular filter and, due to their folate coating, actively bound to FR-α on fibrotic tubules. Unlike passive carriers, these FA-AuNPs were internalised and successfully inhibited intracellular kinase pathways, reversing established tubulointerstitial fibrosis (Chan et al., 2023). Similarly, folate-modified Pluronic F127 micelles loaded with triptolide have been engineered to treat ischemia-reperfusion injury (Qin et al., 2024). By exploiting FR-mediated endocytosis, these micelles delivered the toxic drug triptolide specifically to injured tubules, reducing the required effective dose by 2.9-fold and eliminating the severe hepatotoxicity and reproductive toxicity typically associated with systemic administration (Liu et al., 2019; Yu et al., 2021). While the potential for “off-target” uptake by FR-positive macrophages in other organs remains a consideration, the high density of FRs in the injured kidney provides a favourable therapeutic window for these active targeting strategies (Lin et al., 2024; Haas et al., 2002).

## 3 Strategies for kidney-targeted drug delivery

Having elucidated the physiological barriers and pathological alterations of the kidney, this section examines the specific engineering strategies designed to navigate these obstacles. Current KDDS are broadly categorised into two paradigms: passive targeting, which manipulates the physicochemical properties of carriers (size, charge, and shape) to control their biodistribution within renal compartments; and active targeting, which utilises specific ligands to recognise and bind to receptors, transporters, or enzymes that are uniquely expressed or upregulated in the diseased kidney (Liu et al., 2019; Yu et al., 2021). This section provides a comprehensive classification of emerging kidney-targeted delivery systems, elucidating their design rationales and therapeutic applications (Table 1).

**TABLE 1:** Summary of pharmacological strategies, specific targets, and underlying mechanisms for kidney-targeted drug delivery.

Strategies	Targeting	Mechanism/Rationale
Small-molecule prodrugs & conjugates		
γ-glutamyl modification (e.g., γ-glutamyl-dopa)	Proximal Tubular Epithelial Cells (PTECs)	Enzymatic Activation: Specifically hydrolysed by γ-glutamyl transpeptidase (γ-GT) abundantly expressed on the PTEC brush border to release the active drug locally (Wagner, 2025; Dolman et al., 2010; Rushworth and Megson, 2014)
Hyaluronic Acid modification	Injured PTECs and Activated Macrophages (CD44 receptor)	Ligand-Receptor Binding: Exploits the significant upregulation of the CD44 receptor in inflamed or fibrotic kidneys, using HA as a high-affinity natural ligand (Hu et al., 2018; Sun et al., 2024; Matsushita et al., 2024)
Folic Acid modification	Proximal Tubules & Folate Receptor-positive cells	Receptor-Mediated Endocytosis: Hijacks the high-affinity folate receptor pathway, which is often overexpressed in certain renal pathologies (e.g., polycystic kidney disease) (Low et al., 2008; Chan et al., 2023)
Sugar modification (Glucosyl/Mannosyl derivatives)	Proximal Tubules (SGLT/GLUT transporters)	Transporter-Mediated Uptake: Utilises renal sugar transporters (SGLTs or GLUTs) to transport glucosylated drug conjugates across the cell membrane actively (Suzuki et al., 1999b; Allen et al., 2020; Wang et al., 2020)
Macromolecular & Peptide Carriers		
Low Molecular Weight Chitosan (LMWC)	Proximal Tubular Cells (Megalin receptor)	Megalin-Mediated Endocytosis: Acts as a cationic mucoadhesive polymer that binds avidly to the multiligand receptor megalin, triggering rapid endocytosis into PTECs (Yu et al., 2021; Gao et al., 2014; Williams et al., 2015)
Elastin-like Polypeptide (ELP)	Proximal The Tubules	Biocompatible accumulation: a thermally responsive biopolymer that can be engineered to accumulate in the kidney via endocytosis and prolonged retention (Hong et al., 2024; Peng et al., 2025)
Lysozyme (Low Molecular Weight Protein)	Proximal Tubular Cells (Megalin/Cubilin complex)	Endogenous Reabsorption Pathway: Mimics the natural reabsorption of filtered low-molecular-weight proteins via the megalin/cubilin receptor complex (Natu et al., 2024; Dolman et al., 2012)
G3-C12 Peptide	Proximal Tubular Cells	Phage Display Derived Homing: A specific peptide sequence identified through phage display technology that shows high-affinity binding to renal proximal tubules (Geng et al., 2012)
Nanoparticles (NPs)		
Ultrasmall NPs (Quantum Dots, <10 nm)	Glomerular Basement Membrane (GBM)	Charge & Size Interaction: A small size allows penetration into the GBM, while a surface charge (especially cationic) can promote electrostatic binding to negatively charged proteoglycans (Liang et al., 2016)
Nanoparticles of Defined Size (∼75 ± 25 nm)	Mesangial Cells	Size-Dependent Entrapment: Nanoparticles in this size range pass through endothelial fenestrations but are too large to cross the GBM, leading to passive sequestration in the mesangium (Choi et al., 2011; Dadfar et al., 2020)
Albumin-Conjugated NPs	Podocytes	Albumin-Receptor Binding: Exploits albumin-binding proteins (like gp60) on the podocyte surface to facilitate uptake (Pardhiya et al., 2024)
Mesoscale Nanoparticles (MNP)	Renal Proximal Tubules	Selective Localisation: Larger particles that can localise in the proximal tubule, potentially via mechanisms distinct from standard endocytosis or by exploiting altered vascular permeability (Han et al., 2024)
Functionalized Liposomes		
E-selectin-directed Immunoliposomes	Activated Glomerular Endothelial Cells	Inflammation Targeting: modified with antibodies against E-selectin, an adhesion molecule upregulated on inflamed endothelial cells (Siva et al., 2023; Everts et al., 2003)
Anti-α8 integrin/Anti-Thy1.1 Immunoliposomes	Mesangial Cells	Specific Antigen Recognition: Targets antigens highly expressed on mesangial cells (α8 integrin or Thy1.1) for precise delivery in glomerulonephritis models (Fang et al., 2022; Suana et al., 2011)
Anti-VCAM-1 functionalized liposomes	Activated Podocytes	Inflammation Targeting: Targets Vascular Cell Adhesion Molecule-1 (VCAM-1), which is upregulated on stressed or injured podocytes (Kowalski et al., 2014)

### 3.1 Passive targeting: exploiting physicochemical properties

Passive targeting strategies do not rely on specific molecular recognition but rather on the kidney’s unique anatomical filtration selectivity. By precisely tuning the physical parameters of nanocarriers, therapeutics can be directed to accumulate in the mesangium or traverse the GFB.

#### 3.1.1 Size-dependent accumulation

The hydrodynamic diameter of a nanocarrier is the primary physicochemical determinant of its intrarenal biodistribution and retention profile. The GFB functions as a highly sophisticated biological sieve with a functional molecular weight cutoff of approximately 30–50 kDa, corresponding to a pore size of roughly ∼6 nm (Balbotkina and Kutina, 2023; Cheng et al., 2024). Consequently, ultrasmall nanoparticles, such as quantum dots, dendrimers, and gold clusters with a diameter below 6 nm, undergo rapid renal clearance. These particles freely traverse the endothelial fenestrations and the slit diaphragm, entering the urinary filtrate with minimal interaction with renal cells (Li et al., 2023). While this rapid excretion profile is advantageous for diagnostic imaging agents to minimise long-term toxicity, it poses a significant limitation for therapeutic delivery, as the “residence time” within the kidney is insufficient to facilitate cellular uptake or sustained drug release (Cheng et al., 2024). Recent 2025 pharmacokinetic modelling studies emphasise that, for these ultrasmall carriers, surface modifications (e.g., PEGylation) must be precisely tuned to increase the hydrodynamic volume just enough to delay clearance without preventing filtration entirely, a strategy now being optimised for treating urinary tract infections (Verma et al., 2024).

In contrast, nanoparticles engineered within the 75–100 nm size range exploit a unique anatomical “pathological aperture” known as Mesangial Entrapment. These mesoscale carriers are sufficiently small to pass through fenestrated endothelium (pores 70–90 nm). Still, they are too large to traverse the dense meshwork of the GBM or enter the podocyte slit diaphragm (Choi et al., 2011). As a result, they become passively sequestered within the mesangial space, the central stalk of the glomerulus. This size-dependent localisation is the cornerstone of treating mesangioproliferative diseases, such as diabetic nephropathy and IgA nephropathy (IgAN) (Zhao et al., 2022). A landmark 2024 study demonstrated that 80-nm Celastrol-loaded albumin nanoparticles achieved a 6-fold higher accumulation in mesangial cells compared to 30-nm or 150-nm counterparts (Qu et al., 2024). The endothelial glycocalyx largely excluded the 150-nm particles, while the 30-nm particles were rapidly washed out. This “optimal physiological window” allows targeted delivery of anti-inflammatory agents or immunosuppressants specifically to activated mesangial cells that drive glomerulosclerosis, significantly reducing the systemic toxicity associated with conventional steroid therapy (Qu et al., 2024; Selvaskandan et al., 2024).

However, the “size rule” is not absolute and must be considered in the context of particle deformability and surface chemistry. Under healthy physiological conditions, carriers larger than 150 nm are generally precluded from glomerular entry and are typically cleared by the liver and spleen; yet, recent advances in 2024 have introduced “soft,” deformable nanogels that can squeeze through endothelial fenestrations despite having a resting diameter larger than the pore size (Hou et al., 2024). Crucially, the integrity of this size barrier is dynamically compromised in pathological states. In proteinuric kidney diseases, podocyte effacement and GBM disruption can increase the functional pore size, potentially allowing larger carriers (up to 150 nm) to leak into the tubular lumen and access proximal tubular cells (Meliambro et al., 2024). Therefore, the design of next-generation kidney-targeted therapeutics requires a dynamic understanding of how renal pathology alters these size-dependent barriers. Current clinical translation efforts are focused on creating multistage delivery systems that can change their size in response to the renal microenvironment—for example, shrinking to penetrate deep fibrosis or swelling to enhance retention—thereby maximising the therapeutic index (Shang et al., 2024).

#### 3.1.2 Charge and shape modulation

Surface charge (Zeta potential) acts as a critical “molecular passport” regulating the interaction between nanocarriers and the renal microenvironment. The GFB constitutes a formidable electrostatic shield, characterised by a strong negative charge density arising from sulfated proteoglycans (heparan sulfate) in the GBM and from sialoproteins (podocalyxin) coating the podocytes and the endothelial glycocalyx (Hou et al., 2024). Consequently, cationic nanoparticles (positively charged) exhibit a high affinity for the GFB due to electrostatic attraction. While this facilitates initial binding, it is a double-edged sword. Highly cationic carriers, such as polycationic cyclodextrin or unmodified polyethylenimine (PEI), often cause severe podocyte toxicity, triggering foot process effacement and proteinuria (Dwivedi and Sikarwar, 2025). Furthermore, cationic particles are prone to rapid opsonisation by plasma proteins, leading to swift clearance by the reticuloendothelial system (RES) or non-specific adsorption to the capillary endothelium before they can penetrate the mesangium (Zuckerman et al., 2015; Bennett et al., 2008). Therefore, recent design strategies have shifted towards “charge-reversal” systems that maintain a neutral or negative charge in circulation to ensure safety, but switch to a positive charge only within the acidic tumour or fibrotic microenvironment to facilitate cellular uptake (Shang et al., 2024).

In contrast, anionic nanoparticles (negatively charged) mimic the surface properties of endogenous serum albumin. This anionic surface charge prevents non-specific adsorption to the endothelial glycocalyx, thereby allowing them to penetrate deeper into the glomerular architecture. A seminal study by Liang et al. (ACS Nano) utilising ultrasmall quantum dots (QDs) revealed a distinct charge-dependent fate: while cationic QDs were rapidly excreted, anionic QDs (∼3.7 nm) were retained in the kidney for extended periods, with significant accumulation observed in the mesangial cells (Liang et al., 2016). This “anionic preference” is attributed to the ability of negatively charged carriers to bypass the charge barrier of the GBM while being recognised by scavenger receptors on mesangial cells (Liang et al., 2016; Kodaira et al., 2004). For larger particles targeting the mesangium (75–100 nm), a slight negative surface charge (Zeta potential ∼ −10 to −20 mV) has been shown to optimise retention time by minimising clearance while preventing aggregation (Cheng et al., 2024; Asgeirsdóttir et al., 2008). Thus, surface anionization (e.g., via carboxylation or PEGylation) represents a safer and more effective strategy for long-term drug delivery to the mesangial niche in diseases like diabetic nephropathy.

Beyond size and charge, the nanocarrier’s geometry (shape) exerts a profound yet often overlooked influence on renal hydrodynamics and cellular internalisation. While spherical nanoparticles are the standard, high-aspect-ratio structures such as nanorods, nanotubes, and nanoworms exhibit distinct flow properties. According to fluid dynamics, rod-shaped particles tend to align with the blood flow (laminar flow alignment), which significantly reduces their collision frequency with the vascular wall and minimizes phagocytosis by macrophages compared to spherical particles (Yu et al., 2021). This “stealth” effect prolongs systemic circulation half-life, increasing the probability of renal uptake during repeated passes. Moreover, recent reviews highlight that particle shape influences filtration kinetics; high-aspect-ratio soft nanotubes can “slither” through glomerular pores that would typically exclude rigid spheres of the same volume (Li et al., 2023; Shang et al., 2024). Although shape modulation is technically more challenging to control than size or charge, it offers a frontier for fine-tuning the targeting ratio between the glomerular mesangium (favoured by spheres) and the tubular epithelium (accessible to deformable rods), providing a new dimension for precision nephrology (Balbotkina and Kutina, 2023; Hui et al., 2019).

### 3.2 Active targeting

Unlike passive strategies that rely on stochastic particle accumulation driven by flow dynamics, active targeting employs a “lock-and-key” mechanism. By modifying drug carriers with specific ligands (e.g., peptides, antibodies, carbohydrates), therapeutics can actively recognise and bind to receptors or transporters that are uniquely expressed on renal cells. This approach is indispensable for overcoming the GFB to reach the tubular epithelium and for distinguishing fibrotic lesions from healthy tissue.

#### 3.2.1 Targeting the endocytic pathway

For therapeutic agents smaller than the glomerular filtration threshold (∼6 nm), gaining access to the tubulointerstitium requires “hijacking” the kidney’s intrinsic reabsorption machinery. The proximal tubule’s apical membrane is densely populated with the multi-ligand endocytic receptors megalin (LRP2) and cubilin, which function as a high-capacity “scavenger system” for recovering filtered proteins (Christensen and Birn, 2002). This pathway acts as an ideal “biomimetic hijacking strategy” for active targeting: nanocarriers designed as ligand mimetics bind to these receptors, triggering rapid clathrin-mediated endocytosis and subsequent delivery into the endo-lysosomal compartment. LMWC has emerged as a premier polymeric vector for this strategy due to its inherent cationic charge and specific affinity for megalin (Gao et al., 2014). While early studies focused on intravenous injection, a significant 2024 breakthrough by Zhao et al. demonstrated the feasibility of oral kidney-targeted delivery. They engineered LMWC nanoparticles loaded with the anti-fibrotic agent Salvianolic Acid B, which successfully crossed the intestinal barrier and utilised the megalin pathway to achieve a 4.6-fold increase in renal accumulation compared to free drug in fibrotic mouse models, effectively attenuating collagen deposition (Zhang et al., 2024). Recent quantitative reviews in 2024 further confirmed that optimising the degree of deacetylation and molecular weight of chitosan is critical for maximising megalin binding affinity while minimising systemic clearance (Cheng et al., 2024).

In parallel to synthetic polymers, utilising endogenous proteins or their synthetic peptide mimics represents a highly specific targeting approach. Low-molecular-weight proteins, such as lysozyme (14 kDa), are naturally programmed for near-complete renal sequestration following filtration. Classic strategies involved conjugating anti-inflammatory drugs (e.g., naproxen) to lysozyme via acid-cleavable linkers, ensuring payload release solely within the acidic environment of proximal tubular lysosomes (Haas et al., 1997). However, the use of animal-derived proteins carries risks of immunogenicity and competition with endogenous ligands. To overcome this, recent research has shifted towards synthetic peptide mimics. Short, precisely engineered peptide sequences, such as lysine-glutamic acid repeats [(KKEEE)3K] or cyclic RGD peptides modified to recognise tubular segments, are being developed. A 2024 comprehensive review by Lu et al. highlights that these peptide-drug conjugates (PDCs) offer significant advantages, including defined chemical structures, lower immunogenicity, and the ability to fine-tune megalin binding kinetics, thereby enhancing the therapeutic index for treating tubular diseases (Deng et al., 2026; Lu et al., 2024).

Beyond mere accumulation, this endocytic pathway is currently the most viable route for delivering genetic medicines (siRNA, miRNA, antisense oligonucleotides) to arrest renal fibrosis. Since these nucleic acids require intracellular entry to function, megalin-mediated uptake is essential. Recent 2024 studies have successfully used megalin-targeting polyplexes to deliver siRNA targeting profibrotic genes such as CTGF or Aquaporin-1 (AQP1) directly into injured proximal tubules, achieving gene silencing effects unattainable with systemic administration (Oroojalian et al., 2017).

However, while murine models have provided critical proof-of-concept data, it is imperative to acknowledge that megalin expression levels and receptor recycling kinetics exhibit substantial interspecies divergence. Consequently, the high endocytic capacity observed in rodents may not be directly representative of the human proximal tubule, necessitating rigorous validation in human microphysiological systems or organoids to bridge this translational gap. Furthermore, executing this strategy requires navigating a constrained therapeutic window. As highlighted by Kulkarni et al., the over-administration of nanomaterials can saturate the megalin recycling machinery or exceed the lysosomal degradative capacity, potentially triggering tubular cell stress, autophagic dysfunction, and subsequent nephrotoxicity (Nielsen et al., 2016; Kulkarni and Hussain, 2025).

To circumvent these challenges, the next-generation of endocytosis-targeted vectors must prioritise not only uptake efficiency but also the integration of stimuli-responsive mechanisms for rapid endosomal escape (e.g., pH-sensitive polymers). Such designs are essential to prevent lysosomal sequestration and minimise off-target organelle damage, thereby enhancing both the safety and efficacy of renal genetic therapies (Chen et al., 2020; Liu et al., 2025; Li et al., 2025).

#### 3.2.2 Targeting metabolic enzymes and transporters

While endocytosis handles macromolecules, small-molecule therapeutics can achieve renal specificity by exploiting the unique enzymatic and transport profile of the proximal tubule. The extensive apical brush border of PTECs is densely populated with membrane-bound hydrolases, most notably γ-GT and L-amino acid decarboxylase (Figure 2B). This enzymatic signature provides the rationale for “Enzyme-Activated Prodrugs”: chemical modification masks the drug’s activity in systemic circulation, while renal enzymes act as the specific “trigger” for release. The classic prototype is γ-glutamyl-dopa, a dopamine precursor. Unlike systemic dopamine, which causes hypotension, this prodrug remains inert until hydrolysed by renal γ-GT, releasing active dopamine specifically within the kidney to improve renal hemodynamics (Wilk et al., 1978). In 2024, this concept evolved from simple molecules to smart nanomedicine. Zhang et al. developed a γ-GT-responsive dendrimer-drug conjugate (GSHPD) for treating nephrotic syndrome. This nanosystem utilises a γ-GT-cleavable linker to “lock” the toxic drug triptolide; upon reaching the γ-GT-rich glomerular and tubular epithelium, the linker is cleaved, releasing the payload locally. This “on-site activation” strategy reduced systemic toxicity by orders of magnitude compared to the free drug, representing a paradigm shift in precision toxicology (Chen et al., 2020; Chen et al., 2024).

In parallel with enzymatic activation, “hijacking” the kidney’s nutrient transport machinery provides a high-fidelity entry route. The PT expresses high levels of solute carrier (SLC) transporters to reclaim glucose and vitamins. By conjugating drugs to glucose or glucose analogues (Glucosylation), therapeutics can be recognised as pseudo-substrates by SGLT1/2 (apical) or GLUT1/2 (basolateral) transporters, facilitating active intracellular accumulation (Wagner, 2025). Pioneering work by Suzuki et al., using alkylglucoside vectors, demonstrated that glycosylated vasopressin analogues achieved specific renal membrane retention (Suzuki et al., 1999a). More recently, Lin et al. synthesised prednisolone-glucosamine conjugates that used glucose transporters to achieve a 4.9-fold higher concentration in renal tissue than free prednisolone, effectively mitigating the systemic immunosuppression associated with steroid therapy (Lin et al., 2012b). Current 2025 reviews further suggest that because SGLT2 inhibitors (gliflozins) are now standard of care for CKD, designing conjugates that bind SGLT2 not just for transport but to suppress the NLRP3 inflammasome synergistically represents the next frontier in metabolic targeting (Rabbani et al., 2025; Shang et al., 2024).

Beyond sugars, the FR system provides a dual-targeting mechanism for renal fibrosis. While FR-α is expressed on proximal tubules to reclaim vitamins, its density is significantly upregulated on proliferating, injured tubular cells (e.g., in polycystic kidney disease or fibrosis). Simultaneously, the FR-β isoform is overexpressed on activated, profibrotic macrophages infiltrating the interstitium (Low et al., 2008). This differential expression makes folate an ideal ligand for targeting the entire “fibro-inflammatory unit.” Shi et al. demonstrated that folate-conjugated kinase inhibitors could specifically arrest cyst growth in PKD models by targeting tubular cells (Shi et al., 2019). In a landmark study published in PNAS, researchers engineered sub-10-nm FA-AuNPs. These ultra-small particles were small enough to be filtered but actively bound to FR-α on fibrotic tubules, reversing established tubulointerstitial fibrosis via kinase inhibition (Chan et al., 2023). Similarly, folate-modified polymeric micelles have been used to deliver triptolide to injured kidneys in AKI models, reducing the effective dose by nearly 3-fold and eliminating hepatotoxicity (Qin et al., 2024; Zhao and Guo, 2025).

#### 3.2.3 Targeting the fibrotic microenvironment

In the transition from acute injury to chronic fibrosis, the therapeutic target shifts from the healthy epithelium to the “fibrotic niche”. This microenvironment is characterised by the phenotypic switching of resident cells and the emergence of pathological receptors that are virtually absent in healthy tissue. The most prominent target is the CD44 receptor, which is upregulated more than 10-fold in injured tubular cells and in activated macrophages, mediating cell migration on the extracellular matrix. HA, the natural ligand for CD44, serves as a high-fidelity homing moiety. Recent 2024 advancements have moved beyond simple HA-drug conjugates to multifunctional “theranostic” platforms. For instance, Wang et al. engineered HA-coated CD44-targeted melanin-based nanoparticles. These particles not only specifically accumulated in injured kidneys via CD44-mediated endocytosis but also acted as broad-spectrum antioxidants, scavenging reactive oxygen species (ROS) to protect mitochondria while allowing for photoacoustic imaging of the injury severity (Sun et al., 2024). Similarly, self-assembled HA-bilirubin nanoparticles have been developed to target the CD44-rich inflammatory milieu, where they disintegrate to release bilirubin, neutralise oxidative stress, and arrest the transition from AKI to CKD (Lee et al., 2020; Matsushita et al., 2024).

In parallel with epithelial targeting, interrupting the pericyte-to-myofibroblast transition requires targeting the stromal compartment. Activated myofibroblasts, the primary source of collagen deposition, specifically overexpress integrins, particularly α_v_β_3_ and α_8_β_1_. These integrins activate latent TGF-β1, creating a feed-forward loop of fibrosis. To disrupt this cycle, RGD (Arg-Gly-Asp) peptide-modified nanocarriers have been designed to bind α_v_β_3_ with high affinity. A landmark study used cyclic RGD-functionalized liposomes to deliver small-molecule inhibitors of the YAP/TAZ pathway (a mechanotransduction signal) directly to myofibroblasts. This targeted approach effectively decoupled the cells from the stiff fibrotic matrix, reverting their activated phenotype and promoting tissue regeneration without affecting healthy fibroblasts (Huynh-Cong et al., 2025; Hartner et al., 1999). Furthermore, targeting the platelet-derived growth factor receptor-β (PDGFR-β), which is exclusively upregulated on activated pericytes, has shown promise. PDGFR-β-directed immunoliposomes loaded with interferon-γ have been shown to inhibit myofibroblast proliferation in obstructive nephropathy models selectively (Wang, 2025).

Finally, the chemical composition of the fibrotic microenvironment itself—specifically, the high ROS concentration and acidic pH—can be exploited as a “stimulus” for drug release. ROS-responsive nanocarriers, incorporating thioketal linkers or phenylboronic acid groups, remain stable in the circulation but undergo rapid disassembly upon encountering the oxidative “storm” in the fibrotic kidney. A 2024 study introduced a mitochondria-targeted, ROS-responsive polymer that delivers the antioxidant Mito-TEMPO. Upon internalisation by injured tubules, the high intracellular ROS levels trigger polymer degradation, releasing the payload specifically at the site of mitochondrial damage to prevent necrosis (Wang J. H. et al., 2024). Combining this environmental responsiveness with active ligand targeting creates “logic-gated” systems (e.g., CD44-targeted and ROS-responsive) that maximise therapeutic precision. Recent innovations also include exosome-mimetic nanovesicles engineered to display inflammation-targeting peptides (such as LFA-1) on their surface, allowing them to “utilise endogenous transport” within infiltrating leukocytes to reach the deep renal interstitium (Lu and Huang, 2020; Tian et al., 2021).

## 4 Challenges, future perspectives, and conclusion

### 4.1 Bridging the translational gap

Despite the exponential growth of preclinical successes, the translation of kidney-targeted nanomedicines into clinical practice remains stalled. A primary bottleneck is the physiological discrepancy between species. The vast majority of delivery systems are validated in murine models; however, recent comparative proteomic studies in 2024 reveal significant divergence in the expression density of transporters (e.g., OAT1/3) and the kinetics of megalin recycling between rodents and humans (Zou et al., 2021). Consequently, a carrier that achieves high renal accumulation in a mouse may show poor uptake or unexpected clearance in a human patient. To address this, the field is shifting towards validation in human kidney organoids and microphysiological systems (kidney-on-a-chip), which offer a more predictive model of human renal filtration and reabsorption dynamics than traditional animal studies (Klatt et al., 2025).

Furthermore, the long-term nanotoxicology of non-biodegradable carriers poses a substantial hurdle for regulatory approval. While inorganic materials such as gold nanoparticles or mesoporous silica offer precise size control for mesangial targeting, their potential for chronic retention in the renal parenchyma raises the risk of secondary nephrotoxicity and immunotoxicity. A toxicological review emphasises that even “biocompatible” polymers can induce podocyte stress or tubular inflammation upon repeated administration in CKD patients, whose renal clearance is already compromised. Therefore, future clinical candidates must demonstrate not only targeting specificity but also a defined “clearance mechanism” — ensuring that the carrier can be degraded into non-toxic metabolites or safely excreted after payload delivery (Huang et al., 2024; Chen et al., 2025).

### 4.2 The next horizon

To overcome the immunogenicity and clearance issues of synthetic carriers, the next-generation of therapeutics is pivoting towards bio-inspired engineering, specifically utilising extracellular vesicles. Exosomes derived from mesenchymal stem cells (MSCs) or tubular progenitor cells exhibit innate biocompatibility and the ability to cross biological barriers without triggering immune rejection. Recent 2024 innovations include engineering “hybrid exosomes” that display targeting peptides (such as RGD or KIM-1 ligands) on their surface. These biological vectors combine the intrinsic regenerative properties of extracellular vesicles with the precision of active targeting, offering a “dual-action” therapy that suppresses inflammation while promoting tissue repair (Zhong et al., 2025).

Simultaneously, the complexity of the fibrotic microenvironment demands smarter materials capable of “logic-gated” release. Rather than responding to a single stimulus, emerging nanocarriers are designed to execute Boolean logic (e.g., release drug only if pH is acidic and ROS levels are high). This multi-stimulus responsiveness maximises therapeutic precision, ensuring that potent anti-fibrotics are released strictly within the metabolically stressed “fibrotic niche” while remaining completely inert in healthy nephrons. Such systems are particularly vital for delivering gene-editing tools (CRISPR/Cas9) to correct genetic drivers of nephropathy without off-target editing effects (Li et al., 2019; Asumadu et al., 2025).

## 5 Conclusion

The kidney presents a physiological paradox for drug delivery: its sophisticated filtration and reabsorption machinery evolved to exclude foreign substances, yet these very mechanisms provide the molecular handles for targeted engineering. This review has elucidated that navigating these barriers requires a “function-driven” design philosophy: utilising size (∼75 nm) to access the mesangium, hijacking the megalin pathway for tubular entry, and exploiting pathological receptors (CD44) to treat fibrosis.

Looking forward, the evolution of KDDS must transcend the “one-size-fits-all” approach. The future lies in precision nephrology, where the choice of carrier is dictated by the specific molecular signature of the patient’s disease stage. By integrating advanced theranostic imaging to map receptor expression (e.g., quantifying CD44 levels) with modular carrier design, we can envision a clinical reality in which highly toxic systemic regimens are replaced by precise, local interventions that ultimately halt the progression to end-stage renal disease.
